# Association of TNFRSF19 with a TNF family-based prognostic model and subtypes in gliomas using machine learning

**DOI:** 10.1016/j.heliyon.2024.e28445

**Published:** 2024-03-20

**Authors:** Youwei Guo, Quanwei Zhou, Min Wei, Jianfeng Fan, He Huang

**Affiliations:** a Department of Neurosurgery, Xiangya Hospital, Central South University, Changsha, Hunan Province, China; b National Clinical Research Center for Geriatric Disorders, Xiangya Hospital, Central South University, Changsha, China; c Department of Neurology, Xiangya Hospital, Central South University, Changsha, Hunan Province, China; d The National Key Clinical Specialty, The Engineering Technology Research Center of Education Ministry of China, Guangdong Provincial Key Laboratory on Brain Function Repair and Regeneration, Department of Neurosurgery, Zhujiang Hospital, Southern Medical University, Guangzhou, 510282, China

**Keywords:** Glioma, TNF, Immune cell, Subtype, Prognosis, TNFRSF19

## Abstract

**Purpose:**

TNF family members (TFMs) play a crucial role in different types of cancers, with TNF Receptor Superfamily Member 19 (TNFRSF19) standing out as a particularly important member in this category. Further research is necessary to investigate the potential impact of TFMs on prognosis prediction and to elucidate the function and potential therapeutic targets linked to TNFRSF19 expression in gliomas.

**Methods:**

Three databases provided the data on gene expression and clinical information. Fourteen prognostic members were found through univariate Cox analysis and subsequently utilized to construct TFMs-based model in LASSO and multivariate Cox analyses. TFMs-based subtypes based on the expression profile were identified using an unsupervised clustering method. Machine learning algorithm identified key genes linked to prognostic model and subtype. A sequence of immune infiltrations was evaluated using the ssGSEA and ESTIMATE algorithms. Immunohistochemistry was used to examine the patterns of expression and the clinical significance of TNFRSF19.

**Results:**

Our development of a prognostic model and subtypes based on the TNF family was successful, resulting in accurate predictions of prognosis. The findings indicate that TNFRSF19 exhibited strong performance. Upregulation of TNFRSF19 was correlated with malignant phenotypes and poor prognosis, which was confirmed through immunohistochemistry. TNFRSF19 played a role in reshaping the immunosuppressive microenvironment in gliomas, and multiple drug-targeted TNFRSF19 molecules were identified.

**Conclusions:**

The TMF-based prognostic model and subtype can facilitate treatment decisions for glioma. TNFRSF19 is an outstanding representative of a predictor of prognosis and immunotherapy effect in gliomas.

## 1 Introduction

Glioma is the predominant malignant intracranial neoplasm characterized by a significant fatality rate and unfavorable clinical prognoses [1,2]. Annually, gliomas are diagnosed in approximately 100,000 people worldwide [3]. In the fifth version of the World Health Organization's Classification of Tumors of the Central Nervous System (CNS), gliomas are classified into adult gliomas, pediatric diffuse low-grade gliomas, pediatric diffuse high-grade gliomas, and localized astrocytic gliomas [4]. Despite the standard treatment being used, almost all gliomas relapse [1], particularly in patients with glioblastoma (GBM) [5]. Patients diagnosed with gliomas can expect a median survival time of anywhere between 1 and 15 years, with the duration varying based on the grade of the tumor. GBM is known for its high level of aggressiveness, typically resulting in a median survival time of around 14 months. On the other hand, patients with grade 2 gliomas have a median survival of over 7 years [6]. Thus, it is imperative to build a model capable of predicting the prognosis of glioma and identifying potential therapeutic targets.

At present, several patients with different malignant tumors benefit considerably from immunotherapy [7,8]. Immune checkpoint inhibitors (ICPs) targeting PD-1 and PD-L1 are the main therapeutic choices in glioma immunotherapy [[9], [10], [11]]. Nevertheless, certain patients with glioma do not show satisfactory results with current immunotherapy regimes [12]. The resistance to therapy is believed to stem from the profoundly immunosuppressive and diverse tumor microenvironment of gliomas, which exert a critical influence on the progression of malignancy [13,14].

Increasing evidence suggests that targeting tumor necrosis factor (TNF) receptors is a promising therapeutic strategy for enhancing T-cell responsiveness [15]. TNF components, consisting of 19 TNF ligand groups (TNFSF) and 29 TNF receptor groups (TNFRSF), have the ability to control cellular functions [16]. TNFSF/TNFRSF members have proinflammatory properties that activate the NF-κB pathway to fight pathogens and cancers [17]. Inflammation also promotes the malignant progression of cancers [18]. TNF family members promote inflammation through NF-KB pathway, while TNFSF/TNFRSF family activation can induce cell death and modulate immune responses [19]. Therefore, blocking TNFSF/TNFRSF activity could offer new possibilities for cancer treatment. Therapeutic strategies targeting TNFSF/TNFRSF members like CD40 are being studied in trials for different cancers [20]. TNF-α from nearby tissues can activate the NF-κB pathway to support tumor growth [21]. TNF-α also induces the accumulation of p53 glioma cells, suggesting that p53 may be involved in TNF-α-induced cell death [22].

TNFRSF19, a member of the TNFR superfamily, is associated with increased susceptibility to nasopharyngeal cancer and has been found at elevated levels in various invasive cancers, including colorectal cancer, lung cancer, melanoma, and GBM [[23], [24], [25], [26], [27], [28]]. TNFRSF19 expression in GBM is positively associated with glioma grade and inversely related to clinical outcomes [28]. Increased expression of TNFRSF19 promotes invasion and resistance of GBM cells to TMZ and radiotherapy in both laboratory and living organism settings [29]. Increased expression of TNFRSF19 promotes invasion and resistance of GBM cells to TMZ and radiotherapy in both laboratory and living organism settings.

However, the significance of TNF family members in gliomas is still unclear. Therefore, there is a need to discover new therapeutic targets that could potentially modify the tumor microenvironment either independently or in conjunction with other treatment approaches.In the present investigation, we have effectively delineated the TNF family-derived prognostic model and subtypes, both of which exhibited strong performance in prognostic forecasting.By employing machine learning methods and conducting differential expression analysis, it was found that TNFRSF19 was the only TNF family member that showed significant prognostic precision.Elevated levels of TNFRSF19 were found to be correlated with malignant characteristics and unfavorable prognoses, a finding that was subsequently confirmed through immunohistochemical analysis.

## 2 Materials and methods

### 2.1 Collecting and analyzing data

The research involved 672 individuals with glioma from the TCGA database, along with validation groups from the CGGA dataset (n = 693) and the Rembrandt database (n = 471). The TCGA database can be accessed at https://portal.gdc.cancer.gov/repository, the CGGA dataset at http://www.cgga.org.cn, and the Rembrandt database at https://www.ncbi.nlm.nih.gov/geo. Data was analyzed according to specific criteria, involving the calculation of average mRNA expression data from tumor samples collected from the identical patient and the examination of 677 glioma samples out of a total of 703 files in the TCGA database.Additionally, samples with OS of less than 1 month and those with missing clinical data were excluded from the analysis.

### 2.2 Human specimens and ethical approval

Xiangya Hospital, Central South University provided a glioma tissue microarray containing 31 paired and 124 unpaired glioma tissues, along with 12 normal brain tissues.Every individual gave their consent after being informed, and the actions were conducted in accordance with the Declaration of Helsinki and were approved by the ethics committee of the institution.

### 2.3 Immunohistochemistry (IHC) staining

IHC was conducted utilizing a primary antibody targeting TNFRSF19 (ZEN BIO, China).Two pathologists assessed the results based on the level and proportion of membranous or nuclear reactivity, following previous studies [30]. The protein was mainly found in the nucleus and cytoplasm, showing positive staining in shades of brownish-yellow or tan.High magnification was used to examine four randomly selected fields in order to measure both the total cell count and the count of cells that were positively stained.Staining patterns were assessed by assigning scores based on the percentage of cells stained (ranging from 0 to 4) and the intensity of staining (ranging from 0 to 3). The total score was determined by multiplying the percentage and intensity scores, which were assessed by two independent pathologists.

### 2.4 Development and verification of predictive signatures based on the TNF family

TNF family members were collected from previous research [31] and analyzed using Kaplan-Meier survival curves to determine their correlation with overall survival in three groups.Key individuals were examined using LASSO Cox regression to identify the most important predictive genes in the TCGA group, and a personalized risk assessment was determined according to their mRNA levels. The patients were then categorized into high and low-risk categories based on the median risk scores.The model's prognostic value was evaluated using Kaplan-Meier survival curve analysis.A nomogram was developed using the “rms” package [32]. ROC and calibration curves were used to assess the accuracy and discrimination of risk scores over time with the 'survival ROC' and 'rms' packages in R 3.6.0.

### 2.5 Identification and validation of TNF family-based subtypes in gliomas

The TNF family expression profiles were used to identify subtypes in multiple cohorts using Consensus Cluster Plus analysis [33]. The K-means algorithm was used for 1000 resampling iterations to select samples and probe sets with high variation.

### 2.6 Hub genes associated with TNF family-based subtypes by the root mean squared error (RMSE)

RMSE is a measure used to assess machine learning models, calculated by taking the square root of the average of the differences between predicted and actual values for the entire dataset.In this study, RMSE was employed to identify hub genes among 27 differentially expressed TNF family members.

### 2.7 Immune and stromal cell infiltration analysis

The ESTIMATE method in R was utilized to calculate immune and stromal scores, estimating cell abundance in three cohorts [34]. The ssGSEA method was used to assess 35 cell types, including 33 immune cell types and two stromal cell types, in the TCGA cohort using gene sets from published literature [[35], [36], [37], [38]]. Additionally, the CIBERSORT algorithm [39] and MCP-counter algorithm [40] were employed to quantify the immune cell populations (including CD8^+^ T cells, dendritic cells, natural killer cells, and M2 macrophages) as well as two stromal cell types (endothelial cells and fibroblasts) within the TCGA cohort.

### 2.8 Functional enrichment analysis of TNFRSF19

We investigated the fundamental role of TNFRSF19 by conducting gene set enrichment analysis (GSEA) with GSEA software version 3.0 provided by the Broad Institute (http://www.broadinstitute.org/gsea) [41].

### 2.9 Screening of small-molecule therapeutic drugs acting on TNFRSF19

CellMiner is a platform designed for the examination of molecular and pharmacological information related to the NCI-60 cancer cell line, accessible at https://discover.nci.nih.gov/cellminer/home.do [42].

### 2.10 Statistical analysis

The data from two distinct groups characterized by continuous variables were subjected to analysis through an unpaired Student's t-test within the R software environment. A two-tailed P value below 0.05 was considered to have statistical significance.

## 3 Results

Developing and verifying a predictive model using TNF family proteins in TCGA, CGGA, and Rembrandt datasets.

First, Significant associations with overall survival were found for 37 TNF family members in TCGA, 27 in CGGA, and 28 in Rembrandt cohorts. Seventeen genes were consistently associated with OS in all three cohorts (Table S1). Seventeen overlapping genes linked to OS were identified across three cohorts. A LASSO Cox regression analysis was utilized in the TCGA cohort to identify the 14 most informative genes from a pool of 17 candidate genes, with the aim of developing an optimal TNF family-based risk model. Additionally, the LASSO Cox coefficients for the 14 chosen genes were calculated.The risk score calculation in the TCGA cohort is based on the mRNA levels and LASSO Cox coefficients of 14 genes, with the formula being: risk score = (0.26 * TNFSF14 expression) + (−0.03 * TNFRSF11A expression) + (0.05 * TNFRSF19 expression) + (0.02* TNFRSF10B expression) + (0.08 * TNFRSF14 expression) + (0.0007 * TNFRSF1A expression) + (0.07 * TNFSF4 expression) + (0.05 * CD40 expression) + (0.04 * TNFRSF11B expression) + (−0.3 * TNFRSF10A expression) + (0.14 * CD40LG expression) + (0.002 * NGFR expression) + (0.02 * TNFRSF12A expression) + (0.04 * TNFSF13 expression). Individuals classified as low-risk in the TCGA cohort (n = 307) had significantly longer median survival times than those classified as high-risk (*P* < 0.001, HR 6.34, 95% CI 4.58–8.77). This finding was validated in both the CGGA and Rembrandt groups. Patients classified as high-risk experienced reduced overall survival compared to those classified as low-risk in both the CGGA and Rembrandt groups (*P* < 0.001, HR 2.58, 95% CI 2.10–3.17; *P* < 0.001, HR 2.97, 95% CI 2.34–3.76). Analysis of ROC over time indicated that the model based on TNF family effectively forecasted the OS at 1, 3, and 5 years in the three groups, achieving AUC scores of 0.880, 0.883, and 0.813 in the TCGA group. This suggests that the prognostic model demonstrated effective discrimination between positive and negative prognoses in glioma patients.

### 3.1 The risk score is an independent prognostic factor in the three cohorts

The risk score is a crucial predictor of patient survival in TCGA, CGGA, and Rembrandt cohorts, regardless of other clinical factors, with statistical significance (*P* < 0.05, Fig. 2A–F).Moreover, the ROC analysis validates the predictive precision of this model in contrast to alternative clinical factors. The AUC for the risk score at 60 months was 0.830, while the AUCs for other clinicopathological factors at 60 months varied from 0.510 to 0.803 in the TCGA group, indicating that the risk score outperformed other clinical variables (Fig. 2G).Comparable results were seen in the CGGA and Rembrandt groups when evaluating the risk score (Fig. 2H and I). Our results show that the model's risk score is more accurate in predicting patient outcomes than other clinical factors.

**Fig. 1:**
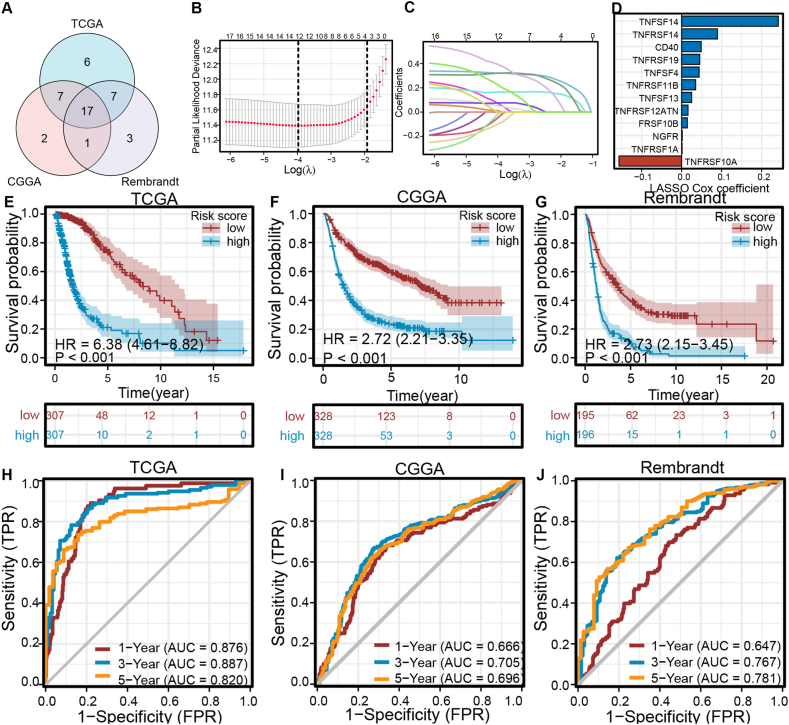
**The development and validation of the 14-signature prognostic model.** (A) TNF family members that are most closely associated with prognosis overlapping in the three cohorts. (B) Partial likelihood deviation distribution of the LASSO coefficient. (C) Partial likelihood deviance revealed by the LASSO regression model. (D) An ensemble of 14 TNF family members with the Cox coefficients. (E–G) Kaplan-Meier curves show the correction between risk scores and the OS of patients in the TCGA (E), CGGA (F), and Rembrandt cohorts (G). The log-rank test was used to determine the statistical differences; *P* < 0.05 was defined as the cut-off criterion. (H–J) Time-dependent ROC analysis at the 1-year, 3-year, and 5-year marks depicted the predicted accuracy of the 14-signature prognostic model in the TCGA cohort (H), CGGA cohort (I), and Rembrandt cohort (J). AUC, area under the curve; ROC, receiver operator characteristic.

**Fig. 2:**
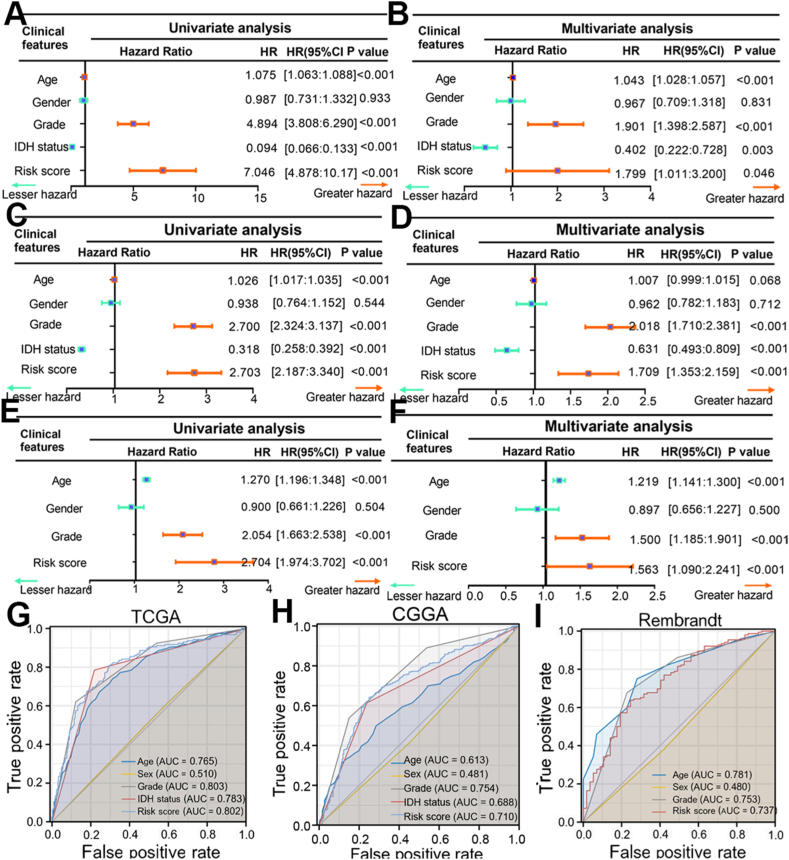
**Risk score performed well as an independent prognostic factor.** (A–F) The univariate and multivariate Cox regression analyses showed that the risk score was a significant variable for OS independent of clinicopathological characteristics in the TCGA (A–B), CGGA (C–D), and Rembrandt cohorts (E–F). (G–I) Time-dependent ROC curves for comparing the prognostic accuracy of risk score with other clinicopathological variables in the TCGA cohort (G), CGGA cohort (H), and Rembrandt cohort (I). Differences with *P* < 0.05 were considered significant. AUC, area under the curve; ROC, receiver operator characteristic; CI, confidence interval; HR, hazard ratio.

### 3.2 Identification and validation of two TNF family-based subtypes in gliomas

**Fig. 3:**
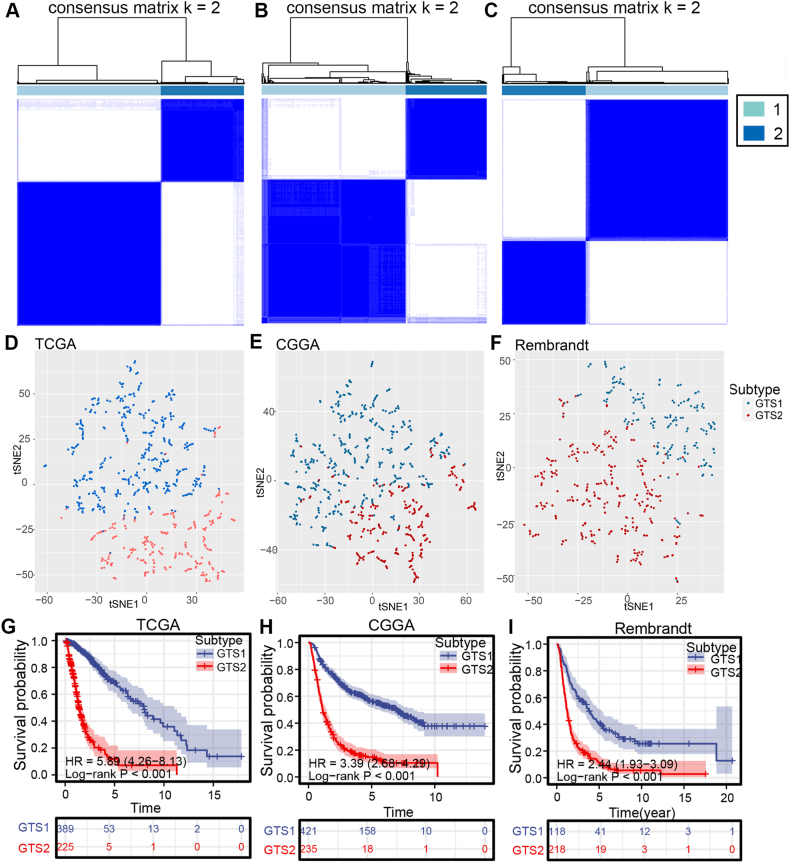
**Identification and validation of TNF family-based subtypes of gliomas in the three cohorts.** (A–C) Heatmaps representing the consensus matrices at k = 2 in the three cohorts. (D–F) The stratification into three subtypes, validated by t-SNE analysis in the three cohorts. Each dot represents a single sample, and each color represents a subtype. (G–I) Survival analysis for the two subtypes (GTS1 and GTS2) of gliomas in the three cohorts. The log-rank test was used to compare the survival curves. A P value < 0.05 was considered statistically significant. OS, overall survival. (For interpretation of the references to color in this figure legend, the reader is referred to the Web version of this article.)

The study utilized the Consensus Cluster Plus methodology to delineate distinct subtypes within a cohort of 672 glioma samples sourced from the TCGA dataset, with classification based on the mRNA expression levels of TNF family genes. The best cluster number (K = 2) was chosen by analyzing the cumulative distribution function (CDF) curve of the consensus matrix (CM) and consensus score shown in Fig. 3A. Consistent findings were also evident in independent validation cohorts from the CGGA and Rembrandt datasets, as illustrated in Fig. 3B and C. We subsequently categorized gliomas into two distinct 1 subtypes, denoted as GTS1 and GTS2. To confirm the distribution of these subtypes, we employed t-SNE for dimension reduction and observed a clear separation of glioma patients into two clusters (Fig. 3D–F). Using a k = 2 classification as mentioned earlier, individuals with GTS2 had notably worse overall survival than those with GTS1 in the TCGA group (HR = 5.89, 95% CI = 4.26 to 8.13, log-rank test *P* < 0.001) (Fig. 3G). A comparable prognostic difference was confirmed in the CGGA cohort (HR = 3.39, 95% CI = 2.68 to 4.29, log-rank test *P* < 0.001) (Fig. 3H) and Rembrandt cohort (HR = 2.44, 95% CI = 1.93 to 3.09, log-rank test *P* < 0.001) (Fig. 3I), showing that individuals with glioma subtype GTS2 had notably reduced OS in comparison to GTS1. Consequently, the identification and validation of the two TNF family-based subtypes of glioma were achieved across the three cohorts.

### 3.3 TNFRSF19 is a hub factor for the TNF family-based prognostic model and subtypes

**Fig. 4:**
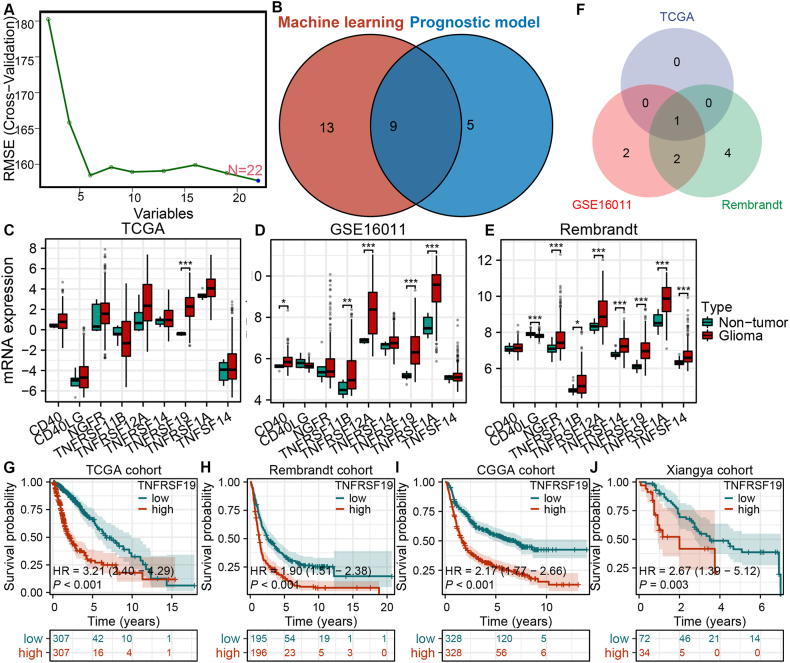
**TNFRSF19 is an important factor in gliomas.** (A) Identification of hub genes using RMSE. (B) Overlapping genes identified by machine learning and LASSO regression analysis. (C–E) mRNA expression of nine shared genes in the TCGA cohort (C), GSE16011 (D), and Rembrandt cohort (E). (F) Overlapping genes identified in the three cohorts. (G–J) Survival analysis of TNFRSF19 in the four cohorts. The log-rank test was used to compare the survival curves, and a P value < 0.05 was considered statistically significant. OS, overall survival.

To identify prognostic markers for clinical use, candidate markers related to different subtypes were mined from participant data by constructing prognostic models. First, the mRNA expression of all TNF family members was compared between two subtypes with gliomas in the TCGA cohort, and 27 differentially expressed TNF family members were identified. Following this, machine learning was used to screen 22 genes from 27 differentially expressed TNF family members (Fig. 4A). Nine genes, namely TNFRSF12A, TNFRSF11B, TNFRSF14, NGFR, CD40, TNFRSF1A, TNFSF14, TNFRSF19, and CD40LG, overlapped with the participator when establishing the TNF family-based prognostic model (Fig. 4B). The clinical significance of gene expression was further elucidated by comparing levels in glioma and non-glioma tissues. TNFRSF19 exhibited significant upregulation in glioma tissues within the TCGA and Rembrandt cohorts (*P* < 0.05, Fig. 4C–F). Kaplan-Meier survival analysis demonstrated a strong association between elevated TNFRSF19 levels and unfavorable clinical outcomes in gliomas across all three cohorts (log-Rank test *P* < 0.05, Fig. 4G–I), consistent with findings from the internal cohort utilizing IHC (log-Rank test *P* < 0.05, Fig. 4J). TNFRSF19 serves as a prognostic marker for adverse outcomes.

### 3.4 Expression pattern of TNFRSF19 in glioma

**Fig. 5:**
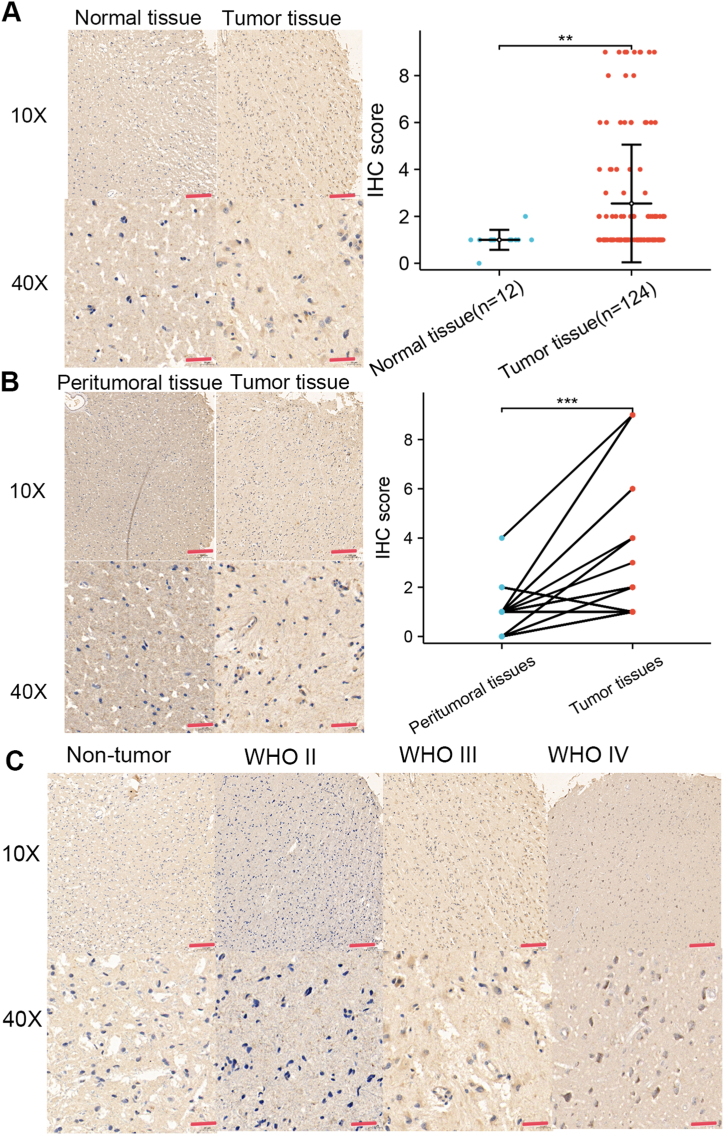
**Expression pattern of TNFRSF19 in gliomas determined using IHC.** (A) The expression of TNFRSF19 in gliomas based on the analysis of glioma and peritumoral tissues using IHC. (B) The expression of TNFRSF19 in gliomas based on the analysis of glioma and peritumoral tissues using IHC. (C) The expression level of TNFRSF19 in gliomas with different WHO grades analyzed by IHC staining. *P* < 0.05 was considered significant.

The study investigated the expression profile of TNFRSF19 in 12 fresh normal and 124 glioma tissues using IHC. As compared to normal tissues and peritumoral tissues, TNFRSF19 was significantly increased in gliomas (*P* < 0.05, Fig. 5A and B). Fig. 5C demonstrated a significant correlation between the increase in TNFRSF19 expression and higher-grade glioma. This finding was further confirmed across four independent cohorts (*P* < 0.05,Supplementary Fig. S1), indicating that elevated TNFRSF19 expression correlates with a more aggressive phenotype in gliomas.

Functional annotation, immune scores, stromal scores, and genomic alteration patterns were compared between glioma samples exhibiting low and high TNFRSF19 expression.

**Fig. 6:**
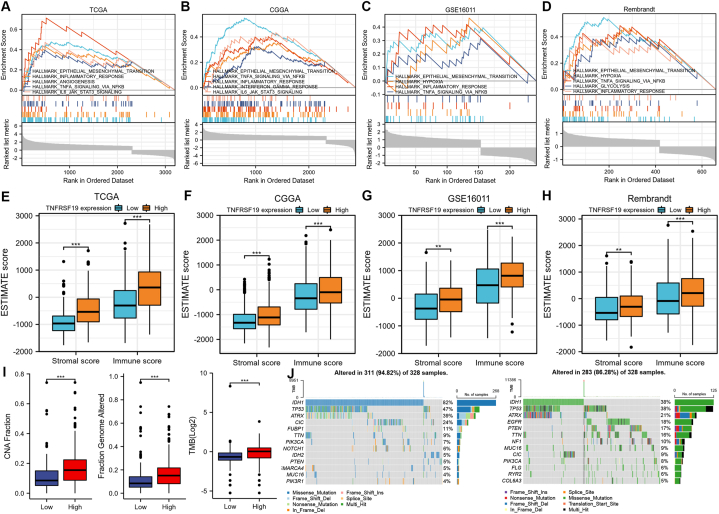
**Comprehensive analyses of enriched pathways, genomic alterations, and immune and stromal scores between glioma samples with low and high expression of TNFRSF19 in gliomas.** (A–D) Gene set enrichment analysis (GSEA) was conducted on gliomas in four cohorts to assess the impact of low and high expression levels of TNFRSF19. Enrichment scores (ES) were considered significant if they met the thresholds of a nominal P-value <0.05 and a false discovery rate (FDR) < 25%. (E–H) The stromal and immune scores were compared between the low and high TNFRSF19 expression groups in the TCGA, CGGA, GSE16011, and Rembrandt cohorts. The significance of the differences was denoted by asterisks on the boxplots (****P < 0.0001). (I) The difference of fraction genome altered, mutation number, and TMB between samples with a low or high expression of TNFRSF19 in the TCGA cohort. (J) A waterfall plot showing the top 15 most frequently mutated genes in glioma samples with a low or high expression of TNFRSF19. The P values are provided above each boxplot with asterisks (**P < 0.01, and ***P < 0.001). GSEA: gene set enrichment analysis; TMB, tumor mutational burden.

The GSEA algorithm was employed to delve deeper into the biological functions within the four cohorts. Our analysis revealed a consistent enrichment of immune response modulation, specifically in terms of “inflammatory response”, “TNF signaling”, and “epithelial-mesenchymal transition”, across all four cohorts (Fig. 6A–D). This finding suggests that immune infiltration may significantly impact biological functions in glioma. Following this, an algorithm known as ESTIMATE was utilized to analyze the immune and stromal scores, which serve as markers for the presence of immune and stromal cells in tumor samples [33]. There was a significant difference in immune and stromal scores between glioma cases with low and high TNFRSF19 expression levels, with higher scores seen in individuals with high TNFRSF19 expression across all four cohorts (*P* < 0.001, Fig. 6E–H). The proportion of genomic alterations, tumor mutational burden, and copy number alteration fraction are linked to tumor immunogenicity [43]. In the TCGA cohort, there was a significant increase in the fraction of genome alteration, tumor mutational burden, and copy number alteration fraction in gliomas exhibiting high expression of TNFRSF19 compared to those with low expression of TNFRSF19 (*P* < 0.05, as shown in Fig. 6I), indicating that gliomas with high TNFRSF19 expression display heightened immunogenicity. Additionally, waterfall plots were utilized to visually represent the genomic alteration patterns in gliomas with differing levels of TNFRSF19 expression (Fig. 6J). IDH1 mutations were found in 82% of gliomas with low TNFRSF19 expression and 38% of gliomas with high TNFRSF19 expression. Gliomas with low TNFRSF19 expression also had higher mutation rates of IDH1, TP53, and ATRX compared to those with high TNFRSF19 expression.

### 3.5 Relationship among TNFRSF19 expression, immune and stromal infiltration, and immune checkpoint expression in glioma

**Fig. 7:**
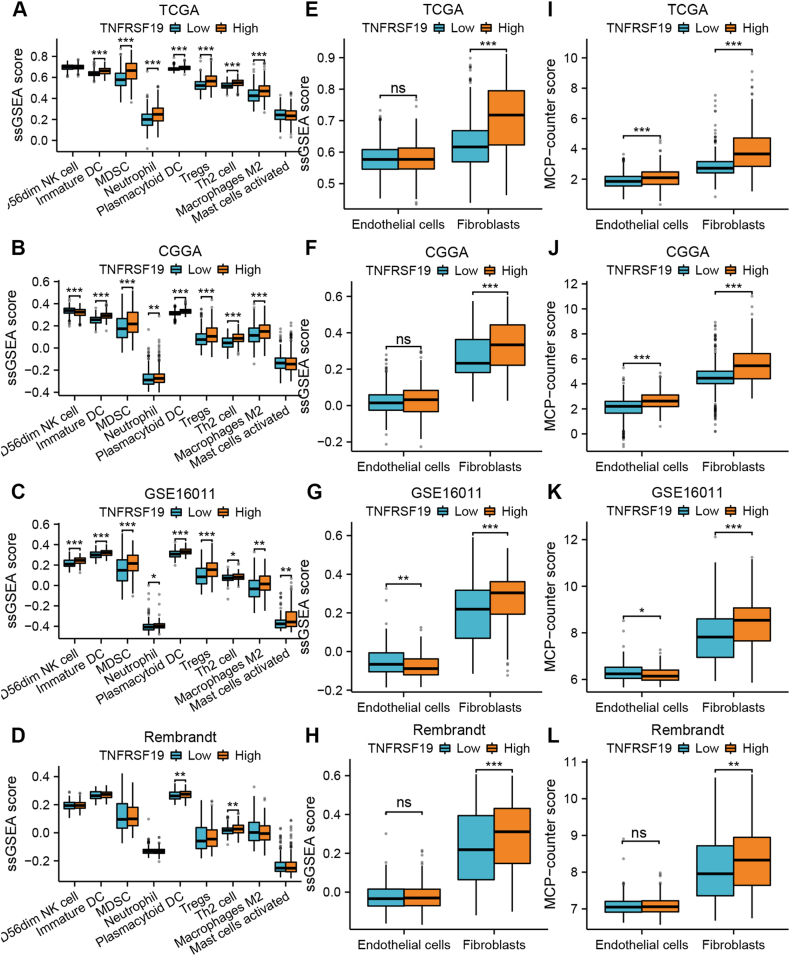
**Correlation between TNFRSF19 expression and immune and stromal infiltration levels in gliomas.** (A–D) The difference in the infiltration of pro-tumor immune cells in gliomas with a low and high TNFRSF19 expression in the TCGA (A), CGGA (B), GSE16011 (C), and Rembrandt cohorts (D), as determined using the ssGSEA method. (E–H) Difference in the infiltration of stromal cells in gliomas with low and high TNFRSF19 expression in the TCGA (E), CGGA (F), GSE16011 (G), and Rembrandt cohorts (H), as determined using the ssGSEA method. (I–L) Difference in the infiltration of stromal cells in gliomas with low and high expression of TNFRSF19 in the TCGA (I), CGGA (J), GSE16011 (K), and Rembrandt cohorts (L), as determined using the MCP-counter method. The statistical difference was compared using the Student's *t*-test. The P values are provided above each boxplot with asterisks (ns represents no significance, *P < 0.05, **P < 0.01, ***P < 0.001).

Our study aimed to explore the role of TNFRSF19 in the immunosuppressive environment of glioma due to the connection found between TNFRSF19 expression and the malignant characteristics.The ssGSEA technique was utilized to examine how TNFRSF19 expression levels correlate with the presence of immune cell populations that promote tumor growth, such as MDSCs, neutrophils, and M2 macrophages.High levels of immunosuppressive cells, excluding CD56dim NK cells, are associated with glioma tumors that have elevated TNFRSF19 expression in various groups of patients (*P* < 0.05, Fig. 7A–C). Significant differences were observed in the occurrence of plasmacytoid DCs and Th2 cells in gliomas with varying levels of TNFRSF19 expression, with higher levels found in tumors with increased TNFRSF19 expression compared to those with decreased TNFRSF19 expression (*P* < 0.05, Fig. 7D). Endothelial cells and fibroblasts in four cohorts were evaluated using the MCP-counter and ssGSEA techniques.Gliomas exhibiting elevated TNFRSF19 levels showed a higher presence of fibroblasts compared to those with lower levels (*P* < 0.05, Fig. 7E-L). Conversely, there was no consistent variance in endothelial cells observed in the TCGA cohort (*P* > 0.05, as depicted in Fig. 7E-L). In summary, the dysregulated expression of TNFRSF19 may play a role in shaping the immunosuppressive microenvironment within gliomas.

**Fig. 8:**
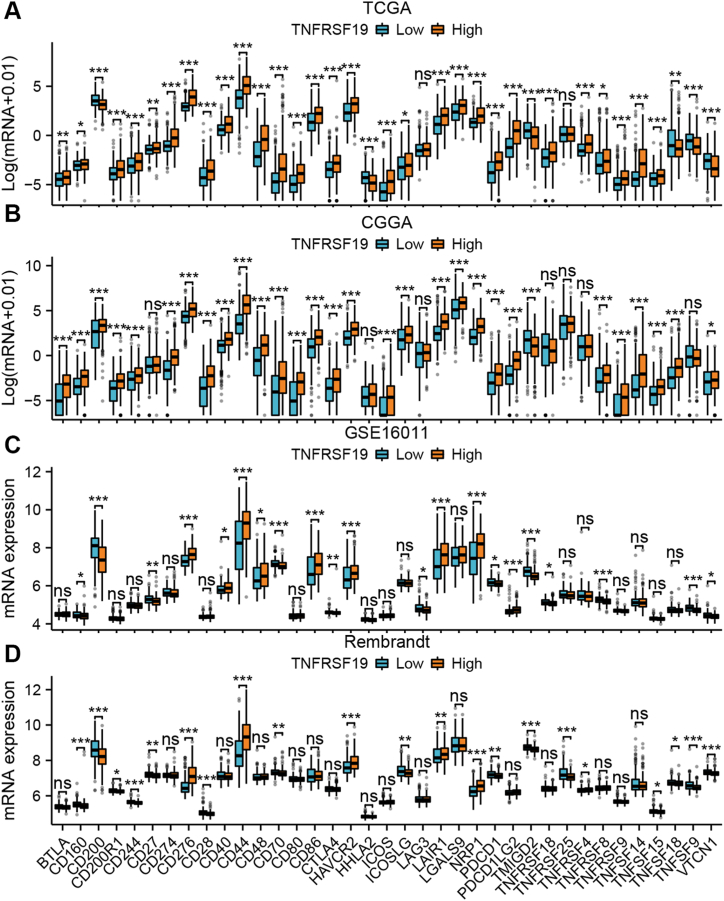
**Correction analysis of TNFRSF19 expression and the mRNA expression of immune checkpoint markers in gliomas in the TCGA (A), CGGA (B), GSE16011 (C), and Rembrandt cohorts (D)**. Statistical differences were compared using the Student's *t*-test, and the P values are provided above each boxplot with asterisks (“ns” represents “no significance”, *P < 0.05, **P < 0.01, ***P < 0.001).

We examined the mRNA expression levels of 37 immune checkpoint genes, specifically PD-L1 and PD-1, in gliomas across four cohorts with varying levels of TNFRSF19 expression. Within the TCGA group, gliomas showing high TNFRSF19 levels exhibited notably increased expression of 28 immune checkpoint proteins, such as PD-L1, and reduced expression of six immune checkpoint genes when compared to those with low TNFRSF19 levels (*P* < 0.05, Fig. 8A). This finding was further confirmed in additional cohorts (*P* < 0.05, Fig. 8B–D). Blocking TNFRSF19 may enhance the effectiveness of immune checkpoint inhibitors.

### 3.6 Screening of small-molecule therapeutic drugs targeting TNFRSF19

**Fig. 9:**
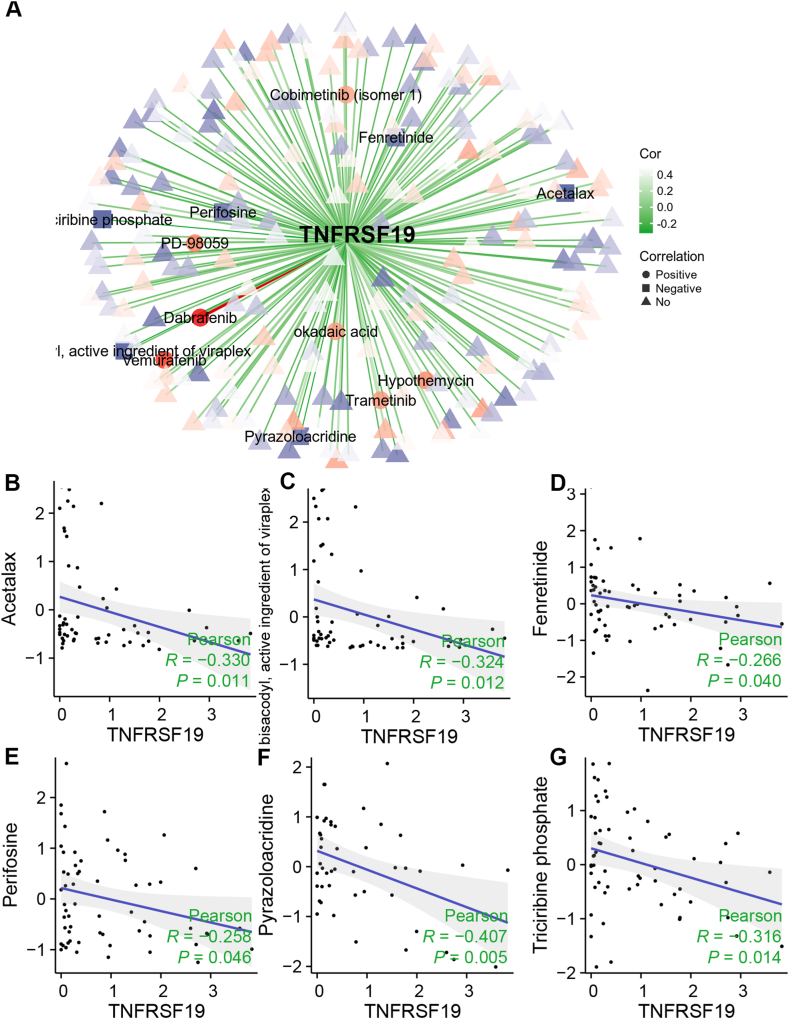
**Prediction of potential drugs targeting TNFRSF19.** (A) The CellMiner database predicts potential drugs targeting TNFRSF19. (B–G) The relationship between drug sensitivity and TNFRSF19 mRNA expression.

Utilizing the CellMiner application, we conducted an analysis of the relationship between drug activity and gene expression in the NCI-60 cell lines, which encompassed glioma cells such as U251 cells. The findings indicated a positive correlation between the expression of TNFRSF19 and the sensitivity of seven drugs, as well as a negative correlation with the sensitivity of six drugs (Fig. 9A). Given the upregulation of TNFRSF19 in gliomas, we further demonstrated a positive association between TNFRSF19 expression and seven drugs (Fig. S2). The results depicted in Fig. S2 indicate a strong correlation between vemurafenib and dabrafenib with TNFRSF19 expression. Conversely, the drug sensitivity of pyrazoloacridine and acetalax demonstrated a negative association with TNFRSF19 mRNA expression (*P* < 0.05, Fig. 9B–G). It is suggested that the combination of these inhibitors with drugs like pyrazoloacridine may enhance the effectiveness of glioma treatment.

## 4 Discussion

Glioma exhibits a significant amount of diversity [44]. While advancements in multimodal treatment have been made in recent years, the efficacy of treatment remains suboptimal, resulting in limited improvement in patient survival [45,46]. Given the intratumoral and intratumor heterogeneity of gliomas, personalized approaches to treatment and prognosis prediction for tumor patients are warranted. Hence, ongoing endeavors are imperative to ascertain tailored prognostic strategies for individualized survival prediction in order to formulate optimal therapeutic and management strategies for patients with varying subtypes of gliomas [45].

Within gliomas, the TNF signaling pathway is recognized as a pro-tumor mechanism that aids in tumor cell evasion of apoptosis and promotes immunosuppression within the tumor microenvironment [19]. The TNF family's capacity to modulate inflammatory reactions is well-documented, leading to the development and testing of antagonists targeting this signaling pathway in clinical trials for inflammatory conditions and cancer [47]. Numerous TNF family members have been implicated in various human diseases, particularly inflammatory conditions and cancers [48]. Members of the TNFSF/TNFRSF exhibit proinflammatory characteristics through the activation of the NF-κB pathway, a central pathway implicated in tumorigenesis and tumor progression. Inflammation has been shown to promote tumor proliferation, metastasis, and angiogenesis in various cancer types. Thus, exploring strategies to regulate TNFSF/TNFRSF activity could offer innovative approaches for cancer therapy. However, the patterns of expression and clinical significance of these individuals in gliomas are not well comprehended at present.

Gliomas exhibit an immunosuppressive tumor microenvironment, prompting the search for dependable molecular biomarkers to assess risk and predict treatment efficacy. The study utilized LASSO-Cox analysis to develop a predictive model using TNF family data from the TCGA group, which was then confirmed in the CGGA and Rembrandt groups (Fig. 1A–G). Furthermore, the risk score was determined to be a significant independent risk factor for unfavorable overall survival in glioma patients, regardless of other clinical characteristics (*P* < 0.05, Fig. 2A–F). The efficacy of the TNF family-based signature was confirmed across multiple independent cohorts (*P* < 0.05, Fig. 2G–I). Notably, two distinct TNF family-based subtypes were identified and validated in all three cohorts, each associated with unique clinical outcomes (Fig. 3G–I). Through the application of machine learning, we have identified nine crucial genes, specifically TNFRSF12A, TNFRSF11B, TNFRSF14, NGFR, CD40, TNFRSF1A, TNFSF14, TNFRSF19, and CD40LG, within the subtype and prognostic model.

TNFRSF12A is implicated in the facilitation of the exogenous apoptotic signaling pathway and the modulation of wound healing. Studies suggest that TNFRSF12A exhibits reduced expression levels in healthy brain tissues, yet demonstrates increased expression in gliomas. Moreover, the increase in TNFRSF11B expression has been demonstrated to enhance the movement and infiltration of glioma cells, while also providing protection against chemotherapy medications in laboratory settings [49]. Originally recognized as a secreted protein and decoy receptor for TRAIL, TNFRSF11B was initially characterized for its interaction with the NF-kB receptor activator, which plays a role in maintaining bone metabolism homeostasis [50]. Recent studies have revealed its implication in the pathogenesis and advancement of diverse human malignancies [51,52]. Furthermore, TNFSF14, known to act as a ligand for TNFRSF11B, has been widely recognized as a significant regulator in immunological and fibrotic disorders [53,54]. Marcos et al. found that the versatility of TNFRSF14 in binding to various ligands in different arrangements results in a diverse range of intrinsic and bidirectional signaling pathways. These pathways play a crucial role in regulating both inflammatory and inhibitory responses, as demonstrated by the inhibitory signals triggered by BTLA engagement with HVEM, ultimately leading to the suppression of T-cell responses [55]. Recent studies have demonstrated that TNFRSF1A-mediated signaling contributes to the promotion of tumor formation in various cancers, including liver cancer, skin cancer, gastric carcinogenesis, and esophageal squamous cell carcinoma (ESCC), as well as the facilitation of cancer cell metastasis [56]. Julia et al. observed that the pharmacological inhibition of NGFR restored T-cell sensitivity in tumor cells [57]. Masashi et al. reported that high expression of CD40/CD40L could potentially serve as a biomarker for improved prognoses in patients with gliomas. Moreover, CD40 agonistic antibodies have demonstrated the ability to induce antitumor responses [58].

TNFRSF19 exhibits tissue-specific expression, with aberrantly elevated levels reported in various invasive cancers including colorectal cancer, lung cancer, melanoma, and GBM [[26], [27], [28]]. Nevertheless, the clinical relevance and expression profile of TNFRSF19 remain unexplored. The findings of our research indicate a significant upregulation of TNFRSF19 in glioma tissues as compared to non-tumor tissues across three cohorts, a result that was further validated in an external set through immunohistochemical analysis (*P* < 0.05, Fig. 5A). Furthermore, TNFRSF19 exhibited elevated expression levels in glioma tissues compared to peritumoral tissues (*P* < 0.05, Fig. 2B). Increased expression of TNFRSF19 was significantly associated with high-grade gliomas (Fig. 2C), suggesting a correlation between TNFRSF19 expression levels and glioma invasiveness. A significant prognostic difference was identified in three cohorts, with high TNFRSF19 expression correlating with a notably shorter overall survival time (log-rank test, *P* < 0.0001, Fig. 4G–I). This prognostic disparity was further confirmed in our own cohort (*P* < 0.05, Fig. 4J), suggesting a potential pivotal role of TNFRSF19 in gliomas. Prior research has also demonstrated the significance of TNFRSF19 in promoting glioblastoma cell invasion and resistance to therapy [59].

Our study on the biological role of TNFRSF19 revealed its involvement in the “inflammatory response”, “TNF/NF-kB signaling pathway”, and “epithelial-mesenchymal transition” across four cohorts (Fig. 6A–D). Previous research has indicated that TNFRSF19 enhances glioma cell survival by activating NF-kB [60]. The findings of the functional enrichment analysis indicate a strong association between TNFRSF19 and immune-specific biological processes and pathways. Gliomas exhibiting elevated TNFRSF19 expression were found to be correlated with heightened levels of immunosuppressive cells across various cohorts, with the exception of CD56dim NK cells (*P* < 0.05, Fig. 7A–C). In gliomas, higher TNFRSF19 expression is associated with increased numbers of plasmacytoid dendritic cells (DCs) and Th2 cells (*P* < 0.05, Fig. 7D).

The research we conducted showed a significant link between elevated TNFRSF19 levels in gliomas and a higher number of fibroblasts when compared to gliomas with lower TNFRSF19 levels (*P* < 0.05, Fig. 7E-L). Conversely, there was no consistent variance in the abundance of endothelial cells within the TCGA cohort (Fig. 7E-L). The results indicate that the uncontrolled TNFRSF19 expression could potentially influence the immunosuppressive environment in gliomas. Furthermore, the observed disparity in overall survival among glioma cases may be linked to immune heterogeneity based on TNFRSF14 expression levels. In addition, the TCGA cohort showed that gliomas with high TNFRSF19 levels had higher amounts of 28 immune checkpoint proteins, such as PD-L1, and lower levels of six immune checkpoint genes compared to those with low TNFRSF14 levels (*P* < 0.05, Fig. 8A). This discovery was subsequently validated in additional cohorts (*P* < 0.05, Fig. 8B and C), suggesting that targeting TNFRSF19 could enhance the efficacy of ICPs. Notably, PD-L1 protein levels and tumor mutational burden (TMB) are established biomarkers for predicting responses to anti-PD-1/PD-L1 therapies [61]. In the TCGA cohort, gliomas exhibiting elevated TNFRSF19 expression demonstrated a significantly higher TMB compared to gliomas with low TNFRSF19 expression, with statistical significance (*P* < 0.05, Fig. 6I). Recent research has demonstrated the potential efficacy of enhancing T-cell responsiveness through the activation of costimulatory receptors within the TNF family, in conjunction with the inhibition of coinhibitory immune checkpoints within the B7-CD28 family [15]. In general, TNFRSF19 demonstrates promise as a target for treatment and a predictor for immunotherapy outcomes in individuals with glioma. TNF inhibitors have demonstrated significant efficacy in reducing inflammation related to various autoimmune diseases [19]. When combined with drugs like pyrazoloacridine, these protein-based agents show promise in enhancing the effectiveness of glioma treatment, as suggested by results obtained through the CellMiner application (Fig. 9). Bioinformatics analysis supported these results, but further experimental confirmation is needed.

## 5 Conclusions

In summary, the TMF-based prognostic model and subtype present a promising tool for predicting glioma prognosis. TNFRSF19 emerges as a notable candidate with potential significance in the pathogenesis of gliomas.

## Ethics declarations

Informed consent was obtained from all patients before they participated. The study protocol underwent approval by the institutional ethics committee, and all procedures were conducted in compliance with the ethical standards outlined in the Declaration of Helsinki.

## Consent for publication

Not applicable.

## Data availability statement

This study analyzed datasets that were accessible to the public. Included in the datasets are (CGGA, http://www.cgga.org.cn/), The Cancer Genome Atlas (TCGA, https://tcga-data.nci.nih.gov/tcga/) databases, GSE16011 database (https://www.ncbi.nlm.nih.gov/geo/query/acc.cgi?acc=GSE16011), and the Rembrandt database (n = 471) (https://caintegrator.nci.nih.gov/rembrandt/).

## Funding

This study was supported by the 10.13039/501100002767Hunan Provincial Science and Technology Department (Grant No. 2021JJ31065).

## CRediT authorship contribution statement

**Youwei Guo:** Writing – review & editing, Writing – original draft, Conceptualization. **Quanwei Zhou:** Writing – review & editing, Writing – original draft, Visualization, Validation, Supervision, Software, Data curation. **Min Wei:** Project administration, Methodology, Investigation, Formal analysis. **Jianfeng Fan:** Methodology, Investigation, Formal analysis. **He Huang:** Writing – review & editing, Writing – original draft, Visualization, Validation, Supervision, Formal analysis, Data curation, Conceptualization.

## Declaration of competing interest

The authors declare that they have no known competing financial interests or personal relationships that could have appeared to influence the work reported in this paper.
