# Supplementary figures and images for: Association of TNFRSF19 with a TNF family-based prognostic model and subtypes in gliomas using machine learning
Source: Heliyon. 2024 Mar 20;10(7):e28445. doi: 10.1016/j.heliyon.2024.e28445 (PMC10979244; doi:10.1016/j.heliyon.2024.e28445)

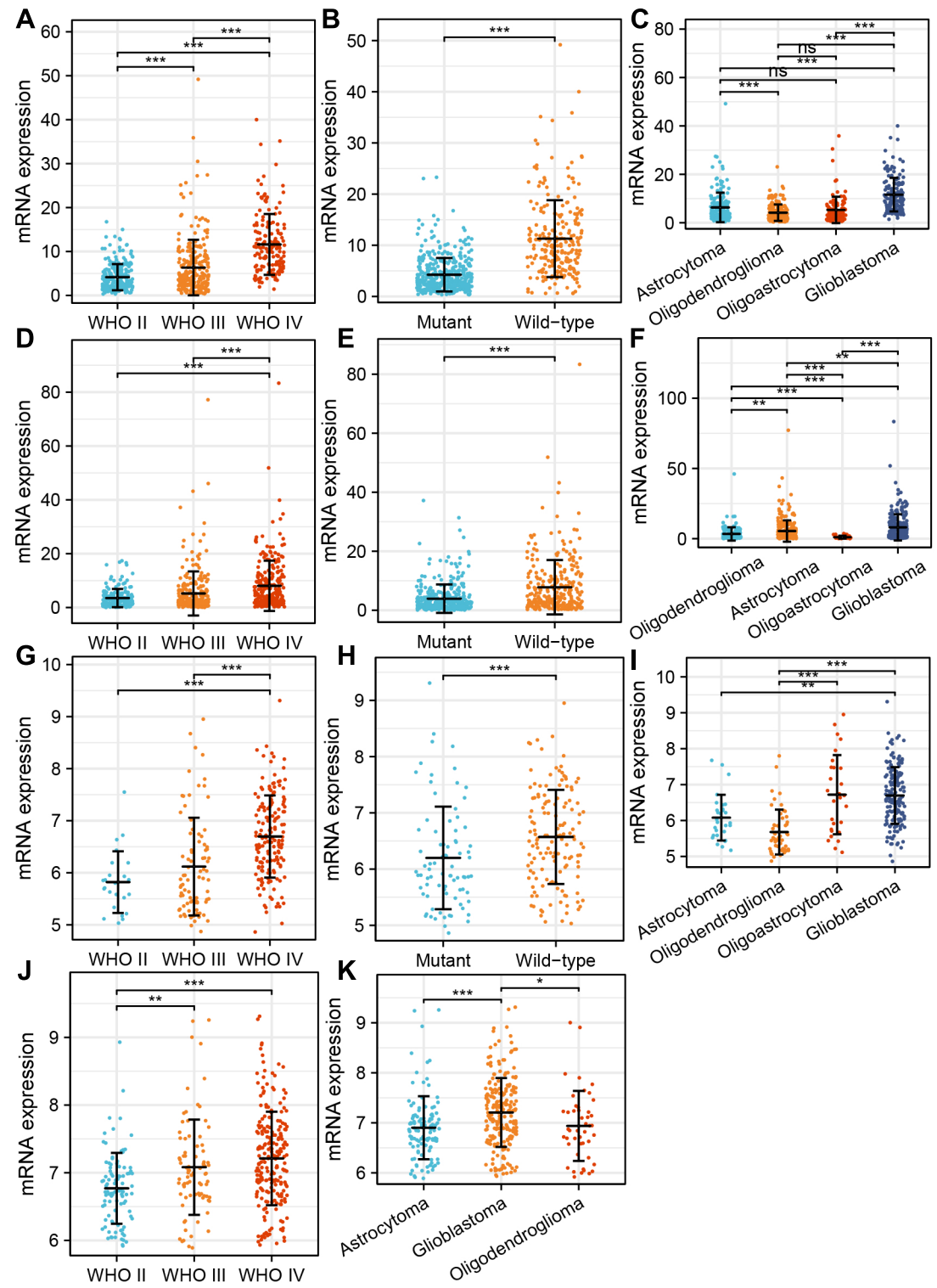

Supplement: Multimedia component 1 [file mmc1.pdf]

**A**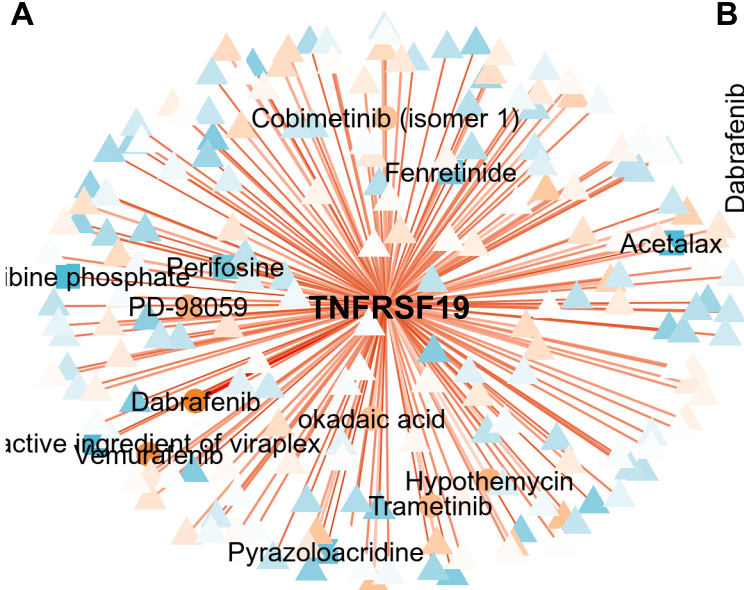**B**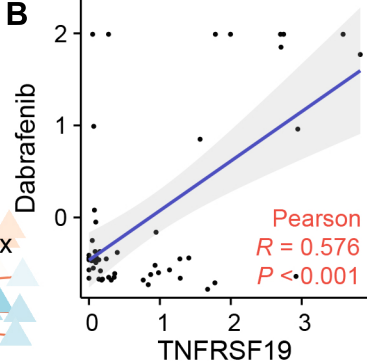**C**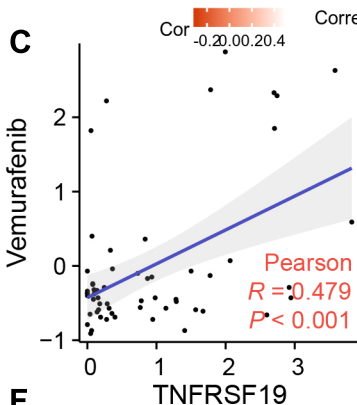**D**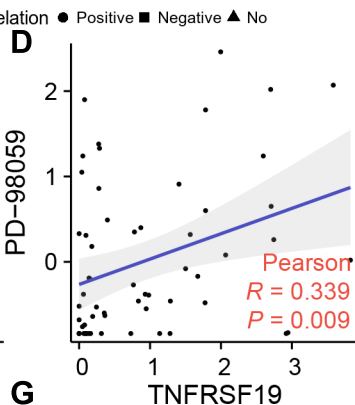**E**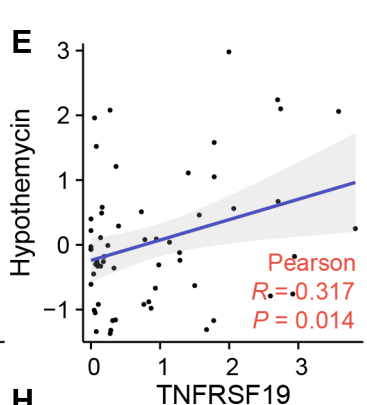**F**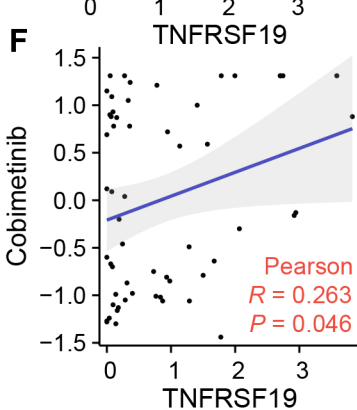**G**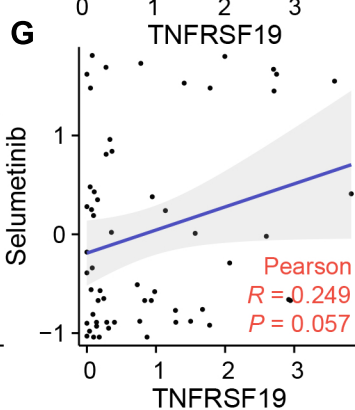**H**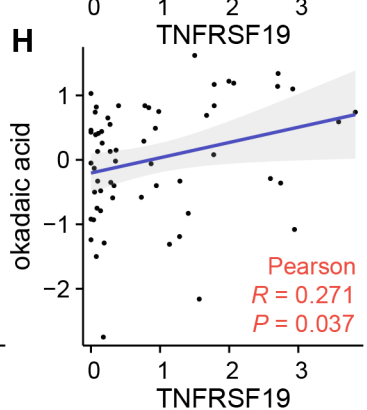

Correlation ● Positive ■ Negative ▲ No

Correlation ● Positive ■ Negative ▲ No

Supplement: Multimedia component 2 [file mmc2.pdf]
